# A comprehensive Beyond-GDP database to accelerate wellbeing, inclusion, and sustainability research

**DOI:** 10.1038/s41597-024-04006-4

**Published:** 2024-10-24

**Authors:** Kedi Liu, Ranran Wang, Paul Behrens, Inge Schrijver, Annegeke Jansen, Irlan A. Rum, Rutger Hoekstra

**Affiliations:** 1 Institute of Environmental Sciences (CML), Leiden University, Leiden, The Netherlands; 2 Oxford Martin School, University of Oxford, Oxford, UK; 3 Universitas Padjadjaran, Bandung, Indonesia

**Keywords:** Interdisciplinary studies, Developing world, Society, Sociology

## Abstract

“Beyond-GDP” metrics are essential for understanding societal progress. Yet despite their importance, these metrics are scattered across various databases, hindering accessibility and interdisciplinary analysis. Addressing this gap, we present the ‘WISE database’ – the first extensive collection of important Beyond-GDP metrics organized by Wellbeing, Inclusion, and Sustainability (WISE) dimensions. The WISE database consolidates data from a variety of sources, including international institutions and academic publications. It encompasses over one million data points across 244 metrics, covering 218 countries and 61 country groupings, from specific social and environmental indicators to the main Beyond-GDP indexes, and is augmented by essential metadata. The data primarily spans from 1995 to 2015, with some metrics extending back to the 19^th^ century. To improve accessibility and data interpretation, a user-friendly online visualization tool has been developed. The WISE database aims to foster a broader view of societal progress, facilitate research on synergies and trade-offs of wellbeing, inclusion, and sustainability, and provide a foundation for interdisciplinary research.

## Background & Summary

The overwhelming focus on Gross Domestic Product (GDP) has led to a societal paradigm focused on economic growth, often at the expense of neglecting wellbeing, socio-economic disparities, and environmental sustainability^1–3^. The term “Beyond-GDP” refers to alternative metrics that present a more comprehensive picture of social progress^4^. These metrics steam from diverse scientific disciplines, such as psychology, sociology, economics, ecology, and philosophy. Notable examples include the Measure of Economic Welfare (MEW) introduced by Nordhaus and Tobin in 1972 from an economic perspective^5^, the first subjective wellbeing surveys by the American Institute of Public Opinion (AIPO) in 1946 which later evolved into the Life Satisfaction (LS) in the World Happiness Report^6^, and Kate Raworth’s Doughnut Economics Framework from 2012 which examines the social foundations and planetary boundaries^7^. International organizations have also developed measurement frameworks and specific indexes to track and promote societal progress. Notable examples include the *Better Life Framework* by the Organisation for Economic Co-operation and Development (OECD)^8^, the Gender Equality Index (GEI) by the European Institute for Gender Equality (EIGE)^9^, and the Human Development Index (HDI) and Sustainable Development Goals (SDGs) by the United Nations (UN)^10,11^.

Though the importance of Beyond-GDP metrics is well-recognized and the data they provide is highly valuable for both policy-making and research, several challenges hinder their effective use. Firstly, there is a lack of consistency in the terminology used to describe these metrics. Terms such as wellbeing, happiness, quality of life, welfare, social progress, or sustainable development are used interchangeably across diverse databases, which could induce confusion and create communication barrier among academics, policy makers, the media, and the public. Secondly, the data for Beyond-GDP metrics are dispersed across numeric databases, each often associated with specific academic groups or international institutions. For example, the World Database of Happiness at the Erasmus University Rotterdam^12^ has focused on subjective wellbeing metrics, such as life satisfaction, which some scholars recognize as a key component of overall sustainable wellbeing^13^. Meanwhile, the World Bank’s Changing Wealth of Nations dataset focusses on wealth accounts, which is also recognized as a measure of sustainability for future wellbeing of nations^14^. The dispersion of Beyond-GDP metrics over a wide variety of databases makes it difficult for researchers to access and be aware of these multiple metrics, thereby preventing a more comprehensive understanding in this field. Additionally, there are inconsistencies in how indicators are labelled across different databases even when they measure similar phenomena. For example, the “Income Inequality” indicator in the Clio-Infra database and the “Equality in Social Foundation” from the Doughnut Economics Framework both use the Gini Index for income inequality, yet they are labeled differently^15,16^. Such discrepancies extend to metadata, including varying country names, which further complicates cross-institutional and interdisciplinary research efforts.

To effectively advance the Beyond-GDP agenda and improve the utilization of existing data tracking societal progress, the establishment of a unified and comprehensive database is imperative^17^. Addressing the challenges of heterogeneous terminology in Beyond-GDP studies, a convergence towards a common framework, referred to as WISE (Wellbeing, Inclusion, and Sustainability), is emerging. This framework synthesizes key findings from significant Beyond-GDP reports: the Brundtland Report^18^, the Stiglitz-Sen-Fitoussi Report^19^, the Conference of European Statisticians Recommendations on Measuring Sustainable Development^20^, and a recent UN report, Valuing What Counts^21^. The WISE framework is also recognized by the OECD’s WISE Centre. Conceptually, the WISE framework categorizes wellbeing into three main dimensions: average current wellbeing (Wellbeing), the distribution of wellbeing within and between countries (Inclusion), and the wellbeing of future generations (Sustainability).

Here, we present the ‘WISE database’ – the first extensive collection of important Beyond-GDP metrics organized by the WISE framework. It comprises more than one million datapoints for 244 metrics from both institutional and academic data sources, covering 218 countries and 61 country groupings, with data mainly extending from the year 1810 to 2023. The dataset includes 210 metrics with at least 20 years of data converge, primarily focusing on the period from 1990 to now. The database comprises two primary types of Beyond-GDP metrics: indexes, which aggregate multiple variables into a single unitless value, and indicators, which measure wellbeing using its own unit independently or in a dashboard such as 231 indicators of Sustainable Development Goals (SDGs). Moreover, the WISE database covers both objective and subjective Beyond-GDP metrics^22^. Every metric in the WISE database has been analyzed and classified according to the WISE framework and is augmented by essential metadata to ensure the consistency and facilitate the utilization. In addition, we developed an online tool to provide open access to the WISE database, allowing users to create various visualizations such as maps, charts, country rankings, and tables (see Usage Note). The infrastructure and methodology behind the WISE database are designed to be flexible, supporting ongoing updates and expansions, and can accommodate research from diverse disciplines such as ecology and sociology, as well as support the efforts of global and national institutions in adopting Beyond-GDP measurements.

## Methods

**Fig. 1:**
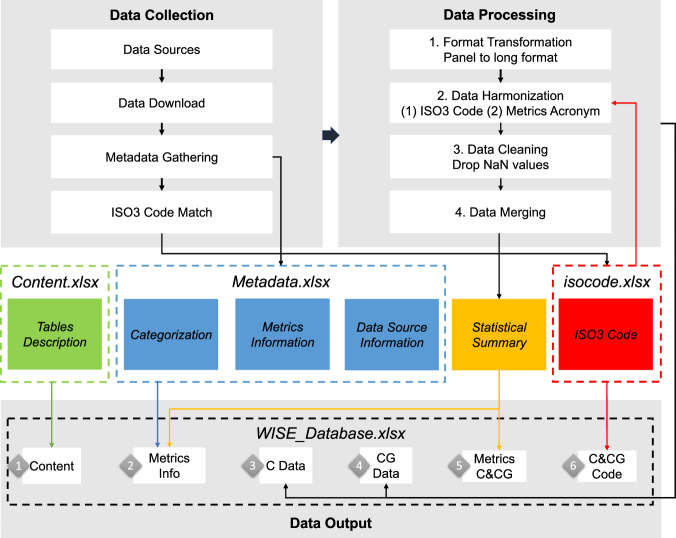
Overview of workflow to build the WISE Database. ISO3 Code is the unified coding names of countries (“C”) and country groupings (“CG”). “Metrics Info” presents the information of metrics.

The WISE database is transparently derived from openly available data sources. The development of the database comprises three main phases: data collection, data processing, and data output as illustrated in Fig. 1, after the metrics selection session. All metric data are gathered from their respective sources and processed to produce a coherent final data output file *WISE_Database.xlsx*.

### Metrics selection

The metric selection for the WISE database is conducted separately for indexes and for indicators. For indexes, we base our selection on a comprehensive review of Beyond-GDP metrics by Jansen *et al*.^22^, which highlights the most influential measurement systems. Out of these indexes, we select only those with data for more than 10 countries using consistent methodologies across studies reporting indexes for one or several countries. This criterion is crucial for enhancing cross-country comparisons, a major aim of the WISE database. Consequently, some well-known metrics, such as Bhutan’s Gross National Happiness (GNH) and the Genuine Progress Indicator (GPI) are excluded due to their limited geographical coverage or inconsistent methodologies.

For indicators, the selection process is centered on creating a comprehensive dashboard that effectively captures the dimensions of well-being, inclusion and sustainability. To ensure an interdisciplinary approach, we base our selection on the Conference of European Statisticians (CES) Recommendations on Measuring Sustainable Development^20^. The framework provided by the CES integrates both theoretical and practical considerations, as well as various initiatives by the UN, OECD, and individual countries. The CES framework itself is largely based on the Stiglitz-Sen-Fitoussi report, an interdisciplinary synthesis of major schools of thought in wellbeing and sustainability, including welfare economics, subjective wellbeing, the capability approach, and planetary boundaries. By aligning available indicators with those recommended in the CES framework, we sourced 73 indicators from the World Development Indicators (WDI) of the World Bank. Additionally, we include indicators directly used to build or are associated with the indexes mentioned earlier, such as the education, health and economic sub-indicators underlying the HDI, to further enrich the WISE database’s dashboard of indicators.

### Data collection

#### Data sources

We compiled all metrics from nine publicly accessible data sources, including both institutional and academic databases (see Table 1). We make no manipulation of the data entries. Detailed information of data sources regarding names, acronyms, reference and source links are documented in the ‘Metrics Info’ page of the final data output WISE database file for reference.

**Table 1:** Summary of Data Sources Included in the WISE Database.

Data Source	Number of metrics	Time Span		Update Frequency
		Start Year	End Year	
Clio-Infra (Composite Measure of Wellbeing^15,25–33^)	10	1500	2016	No updates
De La Escosura, L.P. (Augmented Human Development Index^34^)	10	1870	2020	No updates
European Institute for Gender Equality (Gender Equality Index^9^)	1	2013	2023	Annually
Fanning *et al*. (Doughnut Economics Framework^16^)	51	1992	2015	No updates
United Nations Development Programme (Human Development Index^10^)	36	1990	2022	Annually
Van der Slycken & Bleys. (Index of Sustainable Economic Welfare^35^)	2	1995	2018	No updates
World Bank (Changing Wealth of Nations^14^)	52	1995	2018	2005, 2011, 2018, 2021
World Bank (World Development Indicators^36^)	73	1960	2023	Annually
World Happiness Report (Life Satisfaction^6^)	9	2006	2023	Annually
Total	244			

The corresponding primary metric for each data source is introduced in parentheses. The time span indicates the overall coverage of each data source, note that specific coverage may vary among metrics.

#### Data download

The data were initially downloaded or requested from the publicly available sources listed in Table 2. The specific download options used for each data source are listed in the column ‘Download Option’. For metrics with periodic updates, such as HDI from UNDP and GEI from EIGE, the latest version of data available as of the publication date is included.

**Table 2:** Summary of data download information for all data sources included in the WISE Database.

Data Source	Data Accessibility	Corresponding Website Source	Download Option
Clio-Infra (Composite Measure of Wellbeing^15,25–33^)	Publicly Accessible	https://clio-infra.eu/Indicators/CompositeMeasureofWellbeing.html; https://clio-infra.eu/Indicators/LifeExpectancyatBirthTotal.html; https://clio-infra.eu/Indicators/TotalPopulation.html; https://clio-infra.eu/Indicators/Height.html; https://clio-infra.eu/Indicators/Biodiversitynaturalness.html; https://clio-infra.eu/Indicators/HomicideRates.html; https://clio-infra.eu/Indicators/Polity2Index.html; https://clio-infra.eu/Indicators/GDPperCapita.html; https://clio-infra.eu/Indicators/IncomeInequality.html; https://clio-infra.eu/Indicators/LabourersRealWage.html	“Download Data”: “Compact”
De La Escosura, L.P. (Augmented Human Development Index^34^)	Publicly Accessible	https://frdelpino.es/investigacion/en_gb/world-economy/human-development-world-economy/	“Downloads”: “AHDI countries 1870–2020” and “AHDI regions 1870–2020”
European Institute for Gender Equality (Gender Equality Index^9^)	Publicly Accessible	https://eige.europa.eu/gender-equality-index/2023	“Get the full report and data”: “Gender Equality Index data from 2013 – 2023”
Fanning *et al*. (Doughnut Economics Framework^16^)	Publicly Accessible	https://goodlife.leeds.ac.uk/download-data/	“National Trends”: “Download “The social shortfall and ecological overshoot of nations” national trends data”
United Nations Development Programme (Human Development Index^10^)	Publicly Accessible	https://hdr.undp.org/data-center/documentation-and-downloads	“DATA LINKS”: “All composite indices and components time series (1990–2022)Metadata”
Van der Slycken & Bleys. (Index of Sustainable Economic Welfare^35^)	Publicly Accessible	https://www.sciencedirect.com/science/article/pii/S0921800923003178	“Appendix B. Supplementary data”: “Data”
World Bank (Changing Wealth of Nations^14^)	Publicly Accessible	https://databank.worldbank.org/source/wealth-accounts#	Select “Country”: All; “Series”: All; “Time”: All
World Bank (World Development Indicators^36^)	Publicly Accessible	https://datatopics.worldbank.org/world-development-indicators/	“Access Data”; “Bulk Downloads”: “Excel download”
World Happiness Report (Life Satisfaction^6^)	Publicly Accessible	https://worldhappiness.report/ed/2024/#appendices-and-data	“Appendices & Data”: “DataForTable 2.1”

#### Metadata gathering

We organize the metadata into three parts ‘Categorization’, ‘Metrics Information’, and ‘Data Source Information’ for metrics within the *Metadata.xlsx* file. This step is conducted manually in Excel. Under ‘Categorization’, nine aspects regarding the WISE framework and other fundamental classifications are assigned and documented individually, including ‘Wellbeing’, ‘Inclusion’, ‘Sustainability’, ‘Economy and Society’, ‘Subjective’, ‘Index’, ‘Inclusion Type’, ‘Theme’, and ‘Measurement Type’. In the ‘Metrics Information’, six details of each metric are provided, including the ‘Metric Full Name’, ‘Metric Acronym’, ‘Acronym’, ‘Unit’, ‘Tier’, and ‘Metric Description’. The ‘Metric Full Name’, ‘Unit’ and ‘Metric Description’ are directly obtained from the source per metric, while the ‘Metric Acronym’, ‘Acronym’ and ‘Tier’ are assigned manually by the authors. Lastly, the ‘Data Source Information’ category includes four data fields for each metric: ‘Data Source Full Name’, ‘Data Source Acronym’, ‘Reference’, and ‘Source Link’. These categories are meticulously documented as columns in the excel file, with further elaboration available in Supplementary Information Table S1.

#### ISO3 Code Match

The unified coding information for both country names and country grouping names are provided using the ‘ISO3’ codes, carried out manually in Excel. The names of countries and country grouping are unified for each metric and documented in corresponding worksheets in the excel file *isocode.xlsx*. For clarification, the “countries” or “country” referred in this paper include both nations and territories like Hong Kong or Saint Martin (French part) and are abbreviated as “C” in this work. The “country groupings”, which contain multiple countries based on certain criteria such as geographical locations or economic status, are marked as “CG”. This “C” and “CG” distinction information is documented as ‘Aggregate’ in the WISE database. Notably, we have not created any new country groupings; instead, we have retained and harmonized those present in the original data sources. We applied the ISO-alpha3 code to mark the countries, this is based on the ISO 3-letter codes by International Organization on Standardization^23^. By contrast, the names of country groupings are unified in names instead of letter-codes, this is due to the lack of widely recognized or standardized codes. For example, the country groupings-Europe and Central Asia-is documented as “Europe and Central Asia” and “ZZG.ECA” in the original HDI database but as “Europe & Central Asia” and “ECS” in the WDI database, so the name “Europe and Central Asia” is used as ISO3 code for this country grouping.

### Data processing

The collected source data are processed in four main steps. Each of the nine data sources undergoes processing separately through these steps first before being added to the final data output. This step is conducted in Python, and the corresponding code is available (see code availability).

#### Step 1: Format transformation

Initially, the source data of each metric is transformed into a consistent long-panel format. In this format, each row corresponds to an observation value of one metric for one country or country grouping in a specific year. The metric names and countries or country grouping information are retained for further matching in step 2. As a result, we obtained a dataset in panel form with the original names of metrics and countries or country groupings names from the source data files.

#### Step 2: Data harmonization

In this step, the metric names and country names as well as country grouping names in the original data source files are replaced with unified ISO3 codes and corresponding acronyms, respectively. Firstly, we replace the countries or country groupings names with unified ISO3 code. This is achieved through merging the ISO3 codes and dropping the original names using the information from *isocode.xlsx* file created during the Data Collection phase. For metrics already containing ISO-alpha3 codes in their source data file, such as HDI data, the ISO3 codes for countries (“C” in the ‘Aggregate’) are retained, but the country groupings (“CG” in the ‘Aggregate’) are still replaced with unified names. Metric names are replaced with acronyms in ‘Metrics Information’ of *Metadata.xlsx* in the Data Collection phase. After this step, each metric’s data is structured into five columns: ‘Acronym’, ‘ISO3’, ‘Year’, ‘Value’, and ‘Aggregate’.

#### Step 3: Data cleaning

This step involves eliminating any missing values from the original datasets. All NaN (not a number) data points are removed for each metric. Since the NaN values mostly indicate problems in data collection or entry, removing them can improve the overall quality and reliability of the dataset.

#### Step 4: Data merging

After individual preparation of all metrics following the previous three steps, they are concatenated into a single long dataset. The ‘Unit’ information gathered in the *Metadata.xlsx* is attached. This combined long dataset obtained is further separated into two datasets for countries and country groupings based on the ‘Aggregate’ code “C” or “CG”. Based on the combined long dataset or separated dataset for countries, two ‘Statistical Summary’ containing the descriptive statistics for countries and country groupings or metrics are obtained respectively and documented in data output.

### Data output

As the final step to produce the WISE database, the data and information gathered are reorganized and synthesized into six worksheets in the final data output file *WISE_Database.xlsx*. This step is carried out with Python codes, and the specific procedure to produce each data sheet is detailed specifically as the following:

- “Content” worksheet: To provide a comprehensive overview and clear guidance for users, all columns included in the other five worksheets are listed and described accordingly. Necessary explanations are added as ‘Note’ for certain columns. This information is manually documented in the *Content.xlsx* file and imported as the first “Content” worksheet.
- “Metrics Info” worksheet: This worksheet is constructed by combining all information in the *Metadata.xlsx* obtained in data collection and the ‘Statistical Summary’ gathered after data merging together. Firstly, the ‘Statistical Summary’ obtained here is based on the separated dataset for countries, containing ‘Available Country Count’, ‘Start Year’, ‘End Year’, ‘Scale Min’ and ‘Scale Max’ for each metric. Next, all information in *Metadata.xlsx* is merged with the ‘Statistical Summary’ and exported as the second ‘Metrics Info’ worksheet.
- “C Data” worksheet: The separated dataset for countries in the data merging step is exported as the third “C Data” worksheet.
- “CG Data” worksheet: The separated dataset for country groupings in data merging step is exported as the fourth “CG Data” worksheet.
- “Metrics C&CG” worksheet: the ‘Statistical Summary’ obtained for this worksheet includes ‘Start Year’ and ‘End Year’. This information is based on the combined dataset obtained in data merging, containing both data for countries and country groupings. This information is documented with corresponding ‘ISO3’ and ‘Aggregate’ of each country and country grouping as the fifth “Metrics C&CG” worksheet.
- “C&CG Code” worksheet: This worksheet combines the information of country and country groupings as supplement reference for users. All countries and country groupings contained in the database is listed and linked through ‘ISO3’ code with the “Full List” worksheet in the *isocode.xlsx* file, which contains the ‘Country Name’, ‘ISO Alpha-2 Codes’, ‘ISO Alpha-3 Codes’, ‘ISO Numeric Codes’ and ‘Continent’. The combined results are exported as the sixth “C&CG Code” worksheet.

## Data Records

### WISE_Database.xlsx

**Fig. 2:**
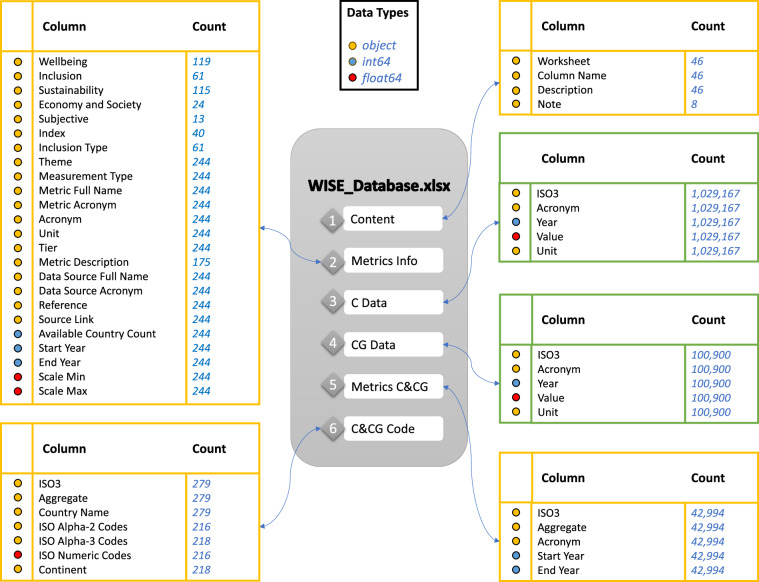
Summary information of the six worksheets contained in *WISE_Database.xlsx*. Four yellow tables document summary information and two green tables include the metrics data values. The data type of each column is illustrated in colored dots.

There are six worksheets in final data output file *WISE_Database.xlsx*. Figure 2 illustrate the column indexes, datatypes, and data counts of the six distinct worksheets described below. *WISE_Database.xlsx* and all source data from nine data sources and the intermediate excel files are publicly available at Figshare repository^24^.

1. “Content” worksheet gives an overview for every column included in all the other five worksheets and provides the detailed description. This worksheet is provided in Supplementary Information Table S1 for reference.
2. “Metrics Info” worksheet provides 24 different attributes information of all metrics included in the database.
3. “C Data” worksheet presents the data for 218 countries.
4. “CG Data” worksheet contains data for 61 different country groupings.
5. “Metrics C&CG” listed the available metrics and its corresponding time coverage for every country and country grouping.
6. “C&CG Code” worksheet contains names and codes information for countries and country groupings contained in the datasets for reference.

### Spatial and temporal coverage

**Fig. 3:**
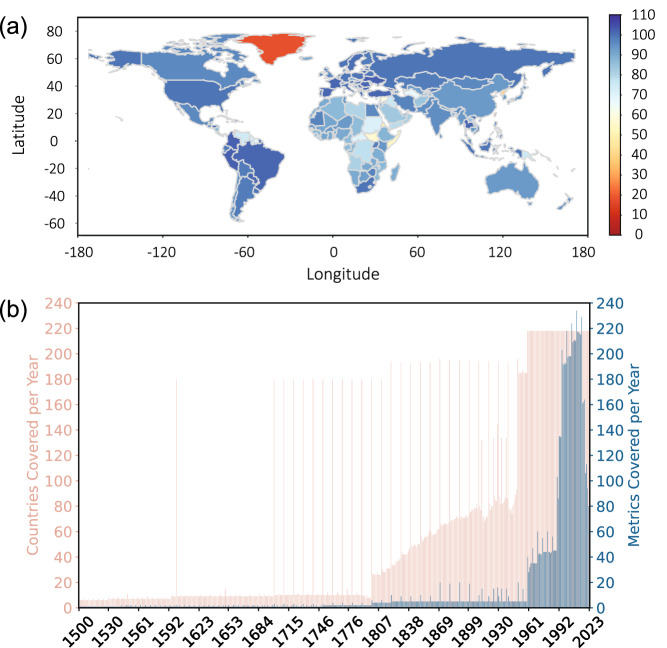
The data coverage of the WISE database. (**a**) Number of metrics covered per country in 2019. (**b**) Number of countries (pink) and metrics (blue) covered per year from 1500 to 2023.

More than one million (1,130,067) data points are included, separated to 1,029,167 data points representing specific countries in “C Data” worksheet and 100,900 data points for country groupings in “CG Data” worksheet. While the specific variables and temporal periods covered may vary from one metric to another, the database collectively spans from 1500 to 2023, and covers overall 218 countries and 61 different country groupings (see Fig. 3). Figure 3(a) depicts the number of metrics covered per country in 2019, a relatively recent year. In this year, most countries are represented by more than eighty metrics, with exceptions observed primarily among African nations. Figure 3(b) showcases a distinct increasing trend in both the number of metrics and countries covered in the WISE database over total time span.

## Technical Validation

Four processes have been implemented to ensure data robustness and reliability throughout the database creation:

### Data source integrity

The metrics incorporated into the WISE database are sourced from recognized international organization or supported by peer-reviewed scholarly publications, with most metrics updated annually.

### ISO3 Code matching

To ensure consistency in geographical identifiers, a detailed matching process has been conducted. This involves manual preliminary cross-checking to identify and correct any duplicates or mismatches between the ISO3 codes and the names used in the original datasets. For example, variations like “Dom. Rep” and “Dominican R” from the AHDI dataset have been unified under the ISO3 code “COD” in the WISE database. The integrity of data was further verified by ensuring the row counts remain consistent the same before and after this ISO3 codes matching process, thus guaranteeing accurate country and country groupings matches.

### Handling of NaN values

NaN (Not a Number) values have been identified and removed from the datasets during the data cleaning step. In this step no valid data points were inadvertently excluded. We have conducted a detailed comparison of NaN and valid data counts before and after processing to verify that all valid data points are retained. To enhance transparency, we report the results in Table 3.

**Table 3:** The summary for NaN and valid data points of each data source in the WISE database.

Data Source	NaN data points	Percentage of NaN data points	Valid data value points		
			C data	CG data	Sum
Clio-Infra (Composite Measure of Wellbeing^15,25–33^)	806482	91.9%	70713	0	70713
De La Escosura, L.P. (Augmented Human Development Index^34^)	6110	14.2%	33970	2880	36850
European Institute for Gender Equality (Gender Equality Index^9^)	0	0.0%	216	8	224
Fanning *et al*. (Doughnut Economics Framework^16^)	15538	8.6%	165686	360	166046
United Nations Development Programme (Human Development Index^10^)	28088	12.8%	180738	11182	191920
Van der Slycken & Bleys. (Index of Sustainable Economic Welfare^35^)	0	0.0%	720	0	720
World Bank (Changing Wealth of Nations^14^)	120	0.1%	182088	0	182088
World Bank (World Development Indicators^36^)	782127	62.9%	374155	86470	460625
World Happiness Report (Life Satisfaction^6^)	386	1.8%	20881	0	20881
Total count:	1638899	59.2%	1029167	100900	1130067

“C data” and “CG data” accounts for the countries and country groupings data respectively. The “Sum” is the number of total valid data points included per data source. The “Total Count” is the counts for final processed dataset.

### Data outliers check

**Fig. 4:**
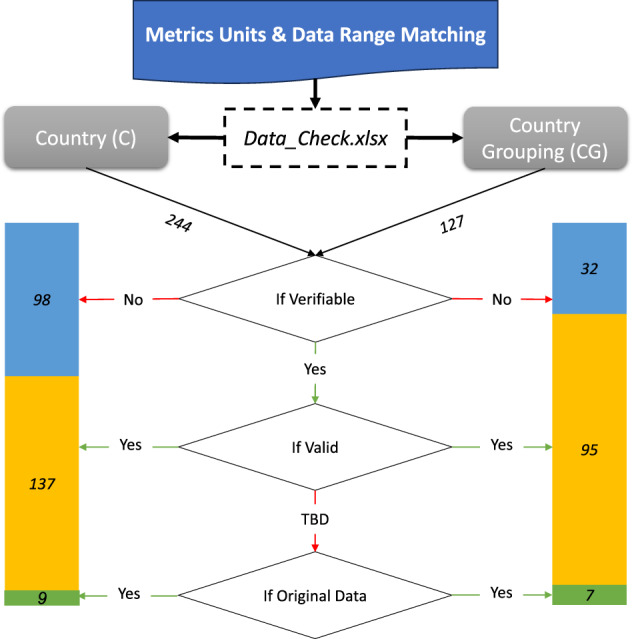
The flowchart representing the process of checking the data outliners. The colored bar charts represent the number of metrics for each category.

We have meticulously checked outliers in the original datasets against expected ranges based on their respective units. This manual check is illustrated in Fig. 4. We initiated this validation process by gathering essential details for each metric, including Metric Name, Acronym, Unit, and Scale (Minimum and Maximum values), and then organizing this information into separate sheets for countries and country groupings (“C” and “CG”) within the file *Data_Check.xlsx*. The validation process then proceeds with three key steps, documented in consecutive columns for each metric. The first column, ‘If verifiable,’ evaluates if the metric’s unit supports reliable data range predictions, categorizing units like indices, percentages, and years as verifiable, and others like monetary units or emissions as not. The subsequent column checks if the data adheres to expected ranges based on the unit, marking data as ‘Yes’ if within expected limits or ‘TBD’ (to be determined) for wider ranges. The final step traces any outliers back to their original sources to confirm their accuracy, ensuring all metrics marked ‘TBD’ are validated and accurate. This comprehensive approach guarantees that the metrics adhere to quality standards and accurately reflect the sourced data.

## Usage Notes

### Inclusion of same metrics from diverse data sources

The WISE database preserves all metrics that measure the same objects but originate from different data sources, rather than treating them as duplicates, to ensure the database’s completeness and facilitate replicability and usability for users. These metrics often serve as components of primary metrics and often exhibit variations in spatial and temporal coverage. For example, Life Expectancy at Birth, a widely studied health metric, appears multiple times across data sources. The primary metrics like HDI and AHDI integrate the Life Expectancy at Birth metric from their corresponding sources which covers contemporary and historical data respectively. To distinguish between these metrics, they are labeled with their respective data source acronyms.

### Online visualization tool

**Fig. 5:**
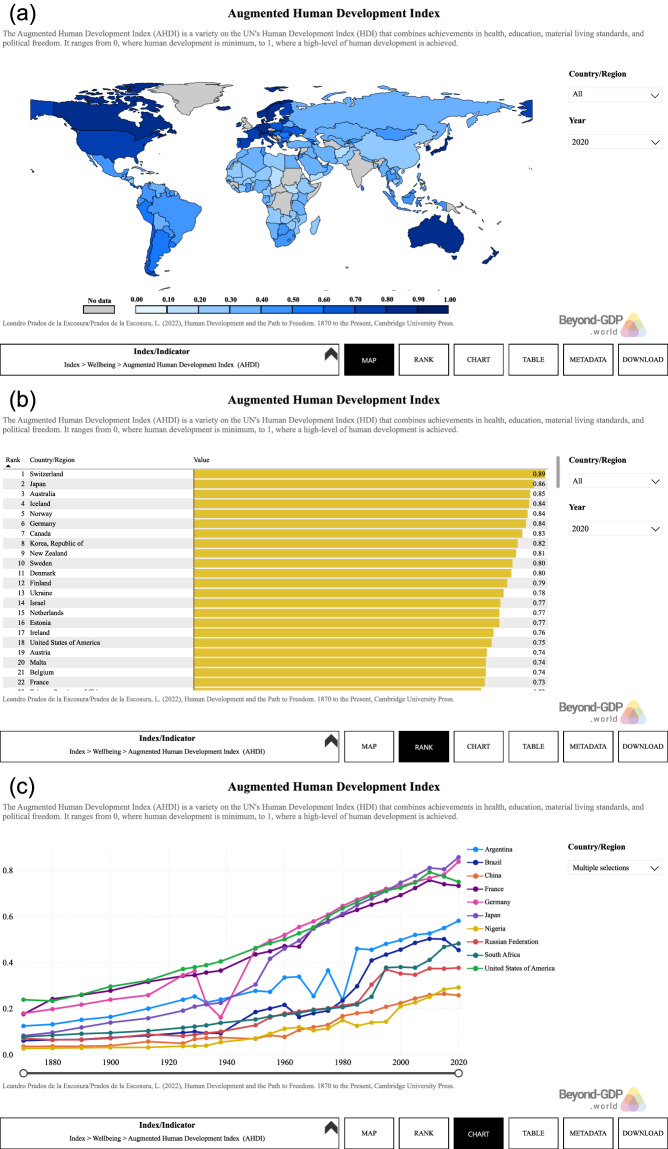
Three main pages of visualization tool for WISE database. The Augmented Human Development Index (AHDI) is displayed here as an example. (**a**) 1. MAP page; (**b**) 2. RANK page; (**c**) 3. CHART page. Other three pages of this visualization tool are 4. TABLE, 5. METADATA and 6. DOWNLOAD. The metrics, countries and time are selectable in the pages 1 to 4.

A data visualization tool based on Microsoft Power BI was developed and made available on https://beyond-gdp.world/wise-database/wise-database (See Fig. 5). The user can select one metric at a time to visualize the data of selected countries and time in formats including world maps, country rankings, tables, and trend charts. The fourth page METADATA contains descriptive information of the selected metric including variable description, institutions/reference, time span and source link. Moreover, the data visualization can be downloaded in PowerPoint format from the fifth page DOWNLOAD.

### Limitations and future work

The WISE database is designed to include the original source data of country level without any data manipulation. Future work could address gaps in time series coverage and explore finer spatial resolutions, such as regional or city levels. Given our focus on consistent methodologies across at least 10 countries, certain important indexes, such as the GPI are excluded. In the future, these indexes could be incorporated as the various scientific groups working on specific Beyond-GDP metrics move towards the harmonization and standardization of their methodologies. The WISE database also offers a structured framework to organize the metrics from diverse scientific disciplines systematically and a foundation to foster future interdisciplinary collaboration.

## Supplementary information

Table S1

## Data Availability

All the codes used to produce the WISE Database are written with Python version 3.10.9 and are openly accessible at GitHub: (https://github.com/kkedliu/WISE-Database.git).
