# Supplementary material for: A comprehensive Beyond-GDP database to accelerate wellbeing, inclusion, and sustainability research
Source: Sci Data. 2024 Oct 24;11:1166. doi: 10.1038/s41597-024-04006-4 (PMC11502754; doi:10.1038/s41597-024-04006-4)
Supplement: Supplementary file 1 — Table S1 [file 41597_2024_4006_MOESM1_ESM.docx]

### Supplementary Information

**Table S1** Description of all metadata available in the WISE database by corresponding worksheets and column names. This information is provided in the “Content” worksheet of data output file *WISE_Database.xlsx*.

| Worksheet | Column Name | Description | Note |
| --- | --- | --- | --- |
| Metrics Info | Wellbeing | The Beyond-GDP metrics relate to measuring the quality of life for the current generation, covering multiple dimensions of prosperity of individuals and society such as happiness, health, education and so on. |  |
| Metrics Info | Inclusion | The Beyond-GDP metrics measure the distribution of wellbeing, assessing the extent to which individuals, regardless of their socioeconomic status, gender, ethnicity, or other characteristics, can participate fully in social, economic, and political activities. |  |
| Metrics Info | Sustainability | The Beyond-GDP metrics reflect the wellbeing of the future, usually measuring the capacity of current societal practices to maintain or improve the wellbeing of future generations while preserving environmental resources. |  |
| Metrics Info | Economy and Society | The Beyond-GDP metrics provide important contextual background information on the socioeconomic perspective but cannot be easily categorized in WIS, such as population, migration, imports and government expenditure or debt. |  |
| Metrics Info | Subjective | The Beyond-GDP metrics include subjective measurements such as surveys, usually based on personal opinions, perceptions, or feelings. |  |
| Metrics Info | Index | Metrics encompass more than one dimension in one number and could be combined from multiple components or cover multiple dimensions. By contrast, an indicator usually only gives a number in one specific measurement aspect. |  |
| Metrics Info | Inclusion Type | Four further detailed types of Inclusion metrics, including 1. distribution (how unevenly resources are distributed throughout a population), 2. ratio (the portion of the population who have resources), 3. threshold (related to the poverty line) and 4. gender (Female or Male). |  |
| Metrics Info | Theme | This information indicates the specific dimensions of societal progress covered by the metric. A total of 21 themes have been identified. | 21 Themes includes: 1. Air Quality 2. Biodiversity 3. Climate Change 4. Consumption 5. Education 6. Energy Resources 7. Financial Capital 8. Governance 9. Health 10. Human Capital 11. Income 12. Knowledge Capital 13. Labour 14. Land 15. Non-energy Resources 16. Physical Capital 17. Population 18. Safety 19. Subjective Wellbeing 20. Summary 21. Water. |
| Metrics Info | Measurement Type | Measurement type indicates the scale and assessment method of the metric of whether it is 1. Level (Aggregate measure which is usually at country-level as a whole) or 2. Average per person (involves the % of selected population in calculation). |  |
| Metrics Info | Metric Full Name | The full name of the metric. |  |
| Metrics Info | Metric Acronym | The acronym of full name of the metric. |  |
| Metrics Info | Acronym | The acronym used to code each metric in this WISE database. | Acronym is constructed with two parts: the Data Source Acronym and Metric Acronym. |
| Metrics Info | Unit | The unit information of the metric. |  |
| Metrics Info | Tier | Hierarchy information of the metric. The tier in to distinguish between primary metrics and their sub-indicators or components (1st to 4th). | The sub tier metrics are usually the components or derivatives for the primary tier metrics. The 1st Tier metrics is the primary metrics, and they are synthesized from the information from the 2nd tier, and this is the same for 3rd and 4th tiers. For metrics that do not have a hierarchical relationship with other metrics, they are all regarded as 1st tier. |
| Metrics Info | Metric Description | A detailed description of the metric (if applicable). |  |
| Metrics Info | Data Source Full Name | The institution or the author of the data source, the corresponding primary metric of each data source is introduced in parenthesis. |  |
| Metrics Info | Data Source Acronym | The acronym of the data source. |  |
| Metrics Info | Reference | The reference information of the publication for the corresponding data source. |  |
| Metrics Info | Source Link | The source link of the data source where the metric is collected from. |  |
| Metrics Info | Available Country Count | Number of available countries (do not include the country groupings) of the metric. |  |
| Metrics Info | Start Year | The earliest available year the metric has data on. |  |
| Metrics Info | End Year | The latest available year the metric has data on. |  |
| Metrics Info | Scale Min | The lower bound of the data values for the metric. |  |
| Metrics Info | Scale Max | The upper bound of the data values for the metric. |  |
| C Data | ISO3 | The ISO3 code of the country for the data point. | ISO3 code for counties are based on ISO 3166-1 alpha-3 code. |
| C Data | Acronym | The acronym of the metric for the data point. |  |
| C Data | Year | The year information for the data point. |  |
| C Data | Value | The value information for the data point. |  |
| C Data | Unit | The unit information for the data point. |  |
| CG Data | ISO3 | The ISO3 code of the country grouping. | ISO3 code for country groupings are based on the unified full name. |
| CG Data | Acronym | The acronym of the metric for the data point. |  |
| CG Data | Year | The year information for the data point. |  |
| CG Data | Value | The value information for the data point. |  |
| CG Data | Unit | The unit information for the data point. |  |
| Metrics C&CG | ISO3 | The ISO3 code of the country or country grouping. |  |
| Metrics C&CG | Aggregate | The information indicating if it's a country ("C") or country grouping ("CG"). |  |
| Metrics C&CG | Acronym | The acronym of the metric. |  |
| Metrics C&CG | Start Year | The earliest available year the metric has data on for the country or country grouping. |  |
| Metrics C&CG | End Year | The latest available year the metric has data on for the country or country grouping. |  |
| C&CG Code | ISO3 | The ISO3 code of the country or country grouping. |  |
| C&CG Code | Aggregate | The information indicating if it's a country ("C") or country grouping ("CG") for the ISO3 code. |  |
| C&CG Code | Country Name | Name of country or country grouping. |  |
| C&CG Code | ISO Alpha-2 Codes | ISO 3166-1 alpha-2 code of the country. | *Kosovo is represented by the assigned code element XK, which is equivalent to ISO 3166-1 alpha-2 code. |
| C&CG Code | ISO Alpha-3 Codes | ISO 3166-1 alpha-3 code of the country. | *Kosovo's assigned code element in the EU is XKX, which is equivalent to ISO 3166-1 alpha-3 code. Assigned code element Channel Islands in the EU is CHI, which is equivalent to ISO 3166-1 alpha-3 code. |
| C&CG Code | ISO Numeric Codes | ISO 3166-1 numeric code of the country. |  |
| C&CG Code | Continent | The continent the country belongs to. | Continents include Africa, Asia, Europe, North America, Oceania and South America. |
